# A qualitative analysis of family experiences across diabetes-specific family functioning types during a family-focused intervention for adults with type 2 diabetes

**DOI:** 10.1007/s10865-025-00594-7

**Published:** 2025-08-14

**Authors:** McKenzie K. Roddy, Erin M. Bergner, Cynthia A. Berg, Lindsay S. Mayberry

**Affiliations:** 1 Department of Medicine, Vanderbilt University Medical Center, Nashville, TN 37203 USA; 2 Center for Health Behavior and Health Education, Vanderbilt University Medical Center, Nashville, TN 37203 USA; 3 Department of Psychology, University of Utah, Salt Lake City, UT 84112 USA; 4 Department of Biomedical Informatics, Vanderbilt University Medical Center, Nashville, TN 37203 USA

**Keywords:** Type 2 diabetes, Family support, Self-management, Qualitative research, Precision medicine, Treatment

## Abstract

Numerous dimensions of family involvement are important for chronic illness management. A recently developed and validated typology of diabetes-specific family functioning organizes these dimensions into four meaningful types (*Collaborative & Helpful*, *Critically Involved*, *Satisfied with Low Involvement,* and *Want More Involvement)*. These types represent patterns of associations across dimensions of family involvement and synthesize these multiple dimensions of functioning into usable categories. The current study had two primary aims: first, to use qualitative data to enhance the quantitative understanding of types; and second, to describe qualitatively participants’ own experiences with their family during a 9 month family-focused intervention (and 6 month follow-up) based on their diabetes-specific family functioning type at enrollment. Adults with type 2 diabetes (T2D) who participated in Family/Friend Activation to Motivate Self-care (FAMS), a family-focused self-care support intervention, were eligible. We recruited 77 participants across types to participate in semi-structured interviews at the completion of the follow-up. We found consistencies across types and differences between types. Regardless of type, harmful family involvement was described, but adults with T2D were hesitant to label it as such. Communication about diabetes and health increased during FAMS, but topics varied across types. Adults with T2D received more support from their families across time, though preference for emotional or instrumental support varied across types. This study qualitatively validated the typology tool paving the way for future use in intervention tailoring.

## Introduction

Robust observational cross-sectional and longitudinal evidence indicates different dimensions of family functioning impact chronic disease self-management (Fort et al., 2020; Rosland et al., 2012). In adults with type 2 diabetes (T2D), specifically, dimensions of helpful family involvement, including autonomy support and emotional support, are associated with better outcomes while dimensions of harmful family involvement, including undermining, control, and criticism, are associated with worse outcomes (Rosland et al., 2012). The effects of harmful involvement on self-care and glycemic management vary by the degree of co-existing helpful involvement (Mayberry & Osborn, 2014). Furthermore, collaboration to address problems/challenges related to T2D can have differential effects depending on the perceptions and needs of the person with T2D. In other words, family functioning and its alignment with the desires of the person with T2D interact to affect outcomes.

This complexity–identified in observational research–may contribute to heterogeneity in effects of family interventions among adults with T2D. Some reviews of family-focused interventions to improve self-management of T2D have found mixed or null results (Baig et al., 2015; Torenholt et al., 2014), whereas others have identified positive effects (Zhang et al., 2022). One hypothesis for differential effects of family interventions is that persons with T2D experience interventions engaging family differently based on their family functioning prior to the intervention. For example, when family functioning is predominantly harmful and mismatched with the desires/needs of the person with T2D, an intervention seeking to increase family involvement may lead to null or iatrogenic effects on outcomes.

Recently, a typology of diabetes-specific family functioning has been developed and validated which organizes nine dimensions of family functioning into four distinct types: *Collaborative & Helpful*, *Critically Involved*, *Satisfied with Low Involvement,* and *Want More Involvement* (Mayberry et al., 2021, 2023). *Collaborative & Helpful* (~ 30%) is notable for high collaborative coping, helpful involvement, autonomy support, satisfaction, and helpfulness with low harmful involvement and perceived criticism; *Critically Involved* (~ 15%) is notable for moderate levels of collaborative coping and autonomy support with very high levels of helpful and harmful involvement and perceived criticism, and moderate effectiveness but low satisfaction; *Satisfied with Low Involvement* (~ 25%) is notable for very low or low scores in all dimensions with no marked dissatisfaction; and *Want More Involvement* (~ 30%) is notable for moderate collaborative coping with low scores across other dimensions (like *Satisfied with Low Involvement*), distinguished by very low effectiveness and satisfaction (Mayberry et al., 2021, 2023). Type is independently associated cross-sectionally and longitudinally with diabetes distress, depressive symptoms, glycemic management, diabetes medication adherence, and diabetes self-efficacy (Mayberry et al., 2021, 2023). Observational studies find patterns of less to more optimal outcomes in this order: *Critically Involved, Want More Involvement, Satisfied with Low Involvement, * and * Collaborative & Helpful* (Mayberry et al., 2021, 2023). Notably, type is not a trait of an individual or a family but rather characterizes a state of family functioning around diabetes management at a specific point in time. Type is constructed of dynamic dimensions of family functioning; therefore, type is likewise dynamic. In the absence of an intervention, ~ 50% change type over 3–6 months (Mayberry et al., 2023). Similar to other “states” such as motivation or self-efficacy to quit smoking, family functioning type may predict intervention engagement and outcomes. This suggests potential for interventions to change family functioning type and for an intervention to be received differently based on family functioning type at intervention initiation.

The recently validated typology creates a new opportunity to investigate participants’ experiences with a family intervention by baseline family functioning type. The Family/Friend Activation to Motivate Self-care (FAMS) intervention targets self-care, self-efficacy, and family involvement (increasing helpful involvement and reducing harmful involvement). FAMS consists of monthly coaching calls to set self-care goals (e.g., increase exercise, improve diet, manage stress) and build skills to manage family involvement (e.g., problem solving, assertive communication) as well as text messages encouraging self-care and monitoring medication adherence and self-care goal completion. Participants have the option to invite a friend or family member to co-participate as a support person who also receives texts about their self-care goals. The recently completed FAMS 2.0 randomized controlled trial (RCT; 2.0 indicates updates and improvements to the FAMS intervention since the pilot RCT; Mayberry et al., 2021) evaluated FAMS versus enhanced treatment as usual on glycemic management, diabetes distress, global well-being, diabetes self-care, diabetes self-efficacy, and family involvement (Mayberry et al., 2022). Compared to control, on average FAMS improved diabetes distress, global well-being, diabetes self-efficacy, helpful family involvement, and dietary behavior; there was not an average effect on glycemic management (Nelson et al., 2023; Roddy et al., 2023). Moderation analyses revealed differences in engagement with and outcomes of the intervention by type in the FAMS 2.0 RCT. Specifically, *Want More Involvement *benefited the most; *Satisfied with Low Involvement* had early improvements that waned; *Collaborative & Helpful* were highly engaged but derived minimal benefits from the intervention; and *Critically Involved* benefitted the least and may have experienced some harm (Roddy et al., 2025). The RCT was preregistered (ClinicalTrials.gov: NCT04347291).

We applied the typology within the FAMS 2.0 RCT to assess type and interviewed participants assigned to FAMS about their experiences to address two aims. As designed, FAMS targets key dimensions of the typology with a goal of increasing helpful involvement and collaboration. All our typology work to date has been quantitative, and we sought to strengthen understanding of the typology with qualitative data representing individuals’ perspectives on their experiences with their family. Additionally, we were interested in qualitatively describing participants’ experiences with family during the intervention based on their diabetes-specific family functioning type at enrollment.

## Methods

We conducted semi-structured interviews with adults with T2D who participated in the FAMS 2.0 RCT and were assigned to the intervention group. As part of the RCT, participants completed assessments, including the typology assessment, at baseline, 6 months (mid intervention), 9 months (post intervention), and 15 months (follow-up). As participants finished their RCT experience, we recruited them purposefully via phone by type assigned at baseline to ensure 15–20 interviews reflecting each type. We also selectively recruited participants for interviews to ensure interviewed participants reflected the clinical and demographic characteristics of the type in the RCT sample. Trained interviewers (male research assistant with limited experience [SM] and female postdoctoral fellow with more experience [MKR]) conducted semi-structured interviews with participants after the RCT 15 -month follow-up, which was approximately 6 months after their completion of the 9- month FAMS intervention. Interviewers had limited relationships with participants (assisted in retention efforts for RCT), and participants’ knowledge of the research came from the informed consent. Informed consent was collected verbally; interviews were conducted over the telephone and lasted approximately 30–45 min. Questions explored participants’ experience with family and friend involvement in their diabetes before, during, and since their FAMS experience, and participants’ experience with FAMS overall and with individual intervention components (e.g., text messages, monthly coaching, invitation to invite a support person to participate in FAMS; see Table 1). Study procedures were approved by the Institutional Review Board (IRB #200398) and participants received $40 for their time.

**Table 1:** Interview questions within structural coding scheme

*Positive FAMS experiences*
What did you like about participating in FAMS?
*Negative FAMS experiences*
What did you dislike about participating in FAMS?
*Support person inclusion*
For this study, you were asked to invite a support person to participate:
What did you think of this request?
Was there anything about your friends/family members or your relationships with them that you considered when thinking about inviting someone?
Did you invite a support person? And if so, tell me how that went for you?
What was it like having that person as a support person in the study?
How did you feel about them getting text messages about your goals?
If you were going to do it again, would you invite the same person?
*FAMS content*
Thinking about the different parts of FAMS, including the text messages, coaching calls, and option to include a support person:
What did you like most about the FAMS program?
What did you like least about the FAMS program?
What would you add to the FAMS program?
What would you remove from the FAMS program?
*Coaching content*
In each coaching call, the coach worked with you to set a self-care goal, discuss your experiences with family/friend involvement, and make a plan to meet your goal and get the support you wanted
What did you like most about the content of the coaching calls?
What did you like least about the content of the coaching calls?
What would you add to the content of the coaching calls?
What would you remove from the coaching calls?
*Family interactions regarding diabetes*
Tell me about how you and your friends/family interacted regarding your diabetes before participating in FAMS
Tell me about how you and your friends/family interacted around your diabetes during FAMS
What have things been like with your family/friends’ involvement in your diabetes since you finished the FAMS?
Tell me how satisfied you have been with these changes or lack of changes in your family/friends’ involvement in your diabetes or health goals
What was the best thing a friend or family member did during your FAMS experience?
What was the worst or least helpful thing a friend or family member did during your FAMS experience?
*Acceptability & desirability of alternative intervention formats & content*
If you could design the best program to meet your diabetes self-care goals, what would you design?
What would you want to learn during the program?
How would you want us to deliver the program?
If you had a chance to do the FAMS program again, would you want to get a version that focused only on your goals and your behaviors or would you keep it as is–with a focus on improving support from your friends and family?

Type was assessed with 37-items from validated measures representing nine dimensions of family functioning including: helpful involvement (emotional support, instrumental support and assistance; Mayberry & Osborn, 2014; Nicklett et al., 2013; Rosland et al., 2012), harmful involvement (nagging, arguing, undermining, and sabotaging behaviors; Leukel et al., 2022; Mayberry & Osborn, 2014; Mayberry et al., 2019; Nicklett et al., 2013), autonomy supportive communication (Lee et al., 2018; Williams et al., 1998), perceived criticism (Karlsen & Bru, 2014), collaborative coping (need for, enjoyment of, and frequency of; (Berg et al., 2011), and match between existing family functioning and adults’ needs (appraisal of it as effective and satisfactory; Song et al., 2012; Tang et al., 2008). We used a multinomial logistic regression equation to calculate participant-specific predicted probabilities for each of the four types. The type with the highest predicted probability was assigned (Mayberry et al., 2023).

### Analyses

Interviews were audio-recorded, transcribed verbatim, and then uploaded in NVivo 12 for coding and analysis (Ltd., 2018). No repeat interviews were conducted, and transcripts were not returned to participants. First, interviews were structurally coded based on the main sections of the interview guide (Table 1). During the structural coding processes, coders reviewed the entire interview transcript for all interviews and allocated data by topic discussed in the interview. After structural coding, we reviewed the data within each structural code and agreed the Family Interactions Regarding Diabetes code contained content with the most generalizable information and most relevant for our questions about experiences by type, rather than information specific to FAMS. If a participant spoke about family interactions outside of the interview questions designated in Table 1, that content was included during the structural coding process. Content in other structural codes was focused largely on feedback about the specific intervention and RCT procedures. Therefore, we decided to focus on data about Family Interactions Regarding Diabetes for thematic analysis. Next, a team of three raters [EB, SM, CG] distributed and reviewed the data coded to Family Interactions Regarding Diabetes, and independently generated notes about emergent themes. The raters then met and discussed their notes to develop an initial thematic codebook for the data pertaining to Family Interactions Regarding Diabetes (Braun & Clarke, 2006). This coding scheme was refined by study authors who had participated in the structural coding and were familiar with the interviews. Then, the final coding scheme was applied iteratively to first three (κ = 90) and then later five (κ = 80) transcripts to establish and confirm inter-rater reliability across 10% of the transcripts. All types were represented across 10% of the transcripts used to evaluate inter-rater reliability. During this process, updates to the codebook were made as needed. Raters then independently thematically coded the rest of the data relevant to Family Interactions Regarding Diabetes, meeting regularly to discuss any questions or resolve any uncertainties.

Next, [MKR & EB] conducted constant comparison analyses by diabetes-specific family functioning types on thematic codes appearing within the topic of Family Interactions Regarding Diabetes (Strauss & Corbin, 1990). Constant comparison procedures compare all data relevant to a particular thematic code with other occurrences of the same thematic code within and across groups (in this case, diabetes-specific family functioning type) to uncover similarities and differences in prevalence as an indicator of salience and dimensions within the themes. We reviewed participants’ narratives about their family and friends’ involvement in their diabetes to identify similarities and differences in thematic codes across and between participants by type. The results presented are based on these constant comparative analyses, whereby similarities and differences in the prevalence and dimensions of the thematic codes by type are summarized.

To enhance the transparency and trustworthiness of our results, we used investigator triangulation (multiple authors were involved in the reading and interpreting of codes and themes) and audit trails (a thorough accounting of each step of the process we undertook) during the coding process. We also triangulated our qualitative results with quantitative research on the typology–both descriptions of the types (Aim 1; Mayberry et al., 2021, 2023) and experiences with the intervention (Aim 2; Mayberry et al., 2021, 2023).

## Results

**Fig. 1:**
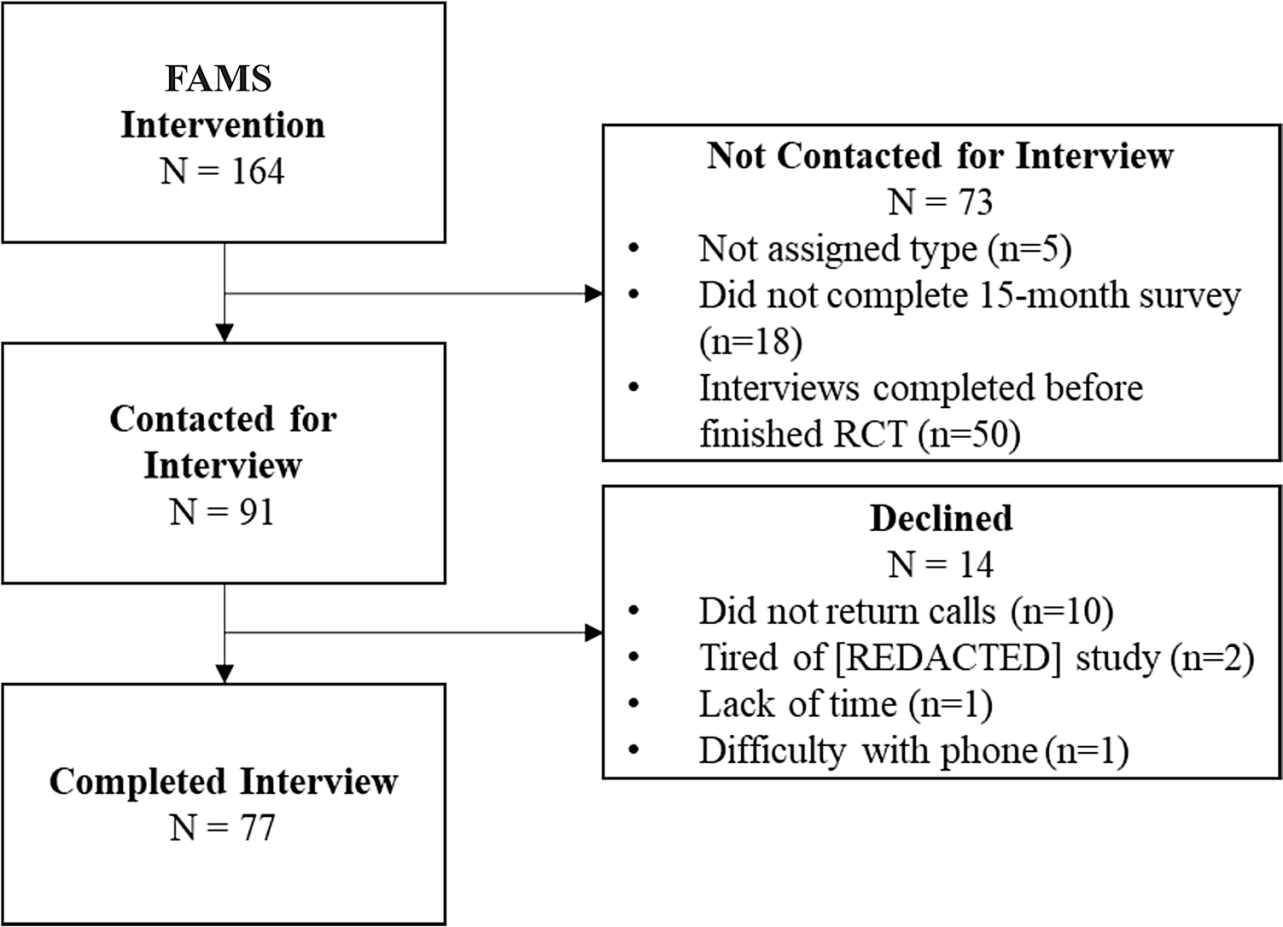
Recruitment for exit interviews was rolling as participants finished the FAMS 2.0 RCT; therefore, some types were fully recruited before some participants were eligible to recruit. If individuals did not complete typology assessment at baseline, they were not assigned a type

Seventy-seven participants completed exit interviews (see Fig. 1). We recruited 20 *Collaborative & Helpful*, 17 *Critically Involved*, 20 *Satisfied with Low Involvement,* and 20 *Want More Involvement*. Participant characteristics are in Table 2*.* The characteristics of participants who completed interviews were consistent with the larger intervention sample by type with regards to gender, age, race and/or ethnicity, and diabetes duration (standardized mean differences < 0.20). In 7 of 28 comparisons (25%), there were differences between the interviewed sample and the RCT sample: interviewed *Collaborative & Helpful* were younger (M = 53.5 vs 56.3 years) and included fewer Hispanic individuals (0% vs 5.2%); interviewed *Critically Involved* included more non-Hispanic Black (41.2% vs 30.4%) and fewer Hispanic (0% vs 4.4%) individuals; interviewed *Satisfied with Low* included more non-Hispanic White (85.0% vs 74.2%) and fewer non-Hispanic Other race(s) (0% vs 6.5%) individuals; and interviewed *Want More Involvement* included more non-Hispanic Black (30.0% vs 19.6%) individuals.

**Table 2:** Characteristics of persons with diabetes at baseline by type

	Mean ± SD or n (%)			
	Collaborative & Helpful (N = 20)	Critically Involved (N = 17)	Satisfied withLowInvolvement (N = 20)	Want More Involvement (N = 20)
*Demographic characteristics*				
Age, years	53.3 ± 11.1	55.4 ± 11.8	58.1 ± 9.4	55.9 ± 12.9
Gender, male	9 (45.0%)	12 (70.6%)	8 (40.0%)	9 (45.0%)
*Race or ethnicity*				
Non-Hispanic white	14 (70.0%)	8 (47.1%)	17 (85.0%)	11 (55.0%)
Non-Hispanic Black	5 (25.0%)	7 (41.2%)	2 (10.0%)	6 (30.0%)
Non-Hispanic other race(s)	1 (5.0%)	2 (11.8%)	0 (0.0%)	0 (0.0%)
Hispanic	0 (0.0%)	0 (0.0%)	1 (5.0%)	3 (15.0%)
*Socioeconomic characteristics*				
Education, years	15.6 ± 2.8	15.9 ± 3.6	15.5 ± 2.2	15.3 ± 2.8
*Annual household income*				
< $35,000	2 (10.0%)	4 (23.5%)	4 (20.0%)	4 (20.0%)
$35,000–$49,999	4 (20.0%)	2 (11.8%)	4 (20.0%)	4 (20.0%)
$50,000–$74,999	3 (15.0%)	4 (23.5%)	1 (5.0%)	4 (20.0%)
$75,000–$99,999	3 (15.0%)	3 (17.6%)	7 (35.0%)	1 (5.0%)
≥ $100,000	5 (25.0%)	4 (23.5%)	4 (20.0%)	6 (30.0%)
*Health insurance*				
Uninsured	0 (0.0%)	0 (0.0%)	1 (5.0%)	0 (0.0%)
Public only	4 (20.0%)	4 (23.5%)	3 (15.0%)	1 (5.0%)
Private	15 (75.0%)	12 (70.6%)	16 (80.0%)	18 (90.0%)
*Clinical characteristics*				
Diabetes duration, years	10.4 ± 7.1	14.3 ± 8.7	9.5 ± 7.3	12.4 ± 7.5
Insulin use	7 (35.0%)	10 (58.8%)	5 (25.0%)	12 (60.0%)
HbA1c (mmol/mol and %)	73 ± 22 8.8 ± 2.0	73 ± 20 8.8 ± 1.9	69 ± 19 8.5 ± 1.7	76 ± 21 9.1 ± 1.9

Annual household income in United States Dollars; Hemoglobin A1c (HbA1c)

Although type was assessed repeatedly over the 15 month RCT, we analyzed by type at baseline because our primary question regarded if different types experienced the intervention differently. Additionally, using baseline type aligns with the quantitative analyses that found differences in engagement and outcomes by baseline type (Roddy et al., 2025). Over the 15 month RCT, over half of participants (52%) were stable in their type (≥ 2 of 3 follow-ups were same type as baseline). Around one quarter (26%) of participants who were another type at baseline moved into and were stable in *Collaborative & Helpful*. The remaining 21% moved to and were stable in a type other than *Collaborative & Helpful* and their baseline type or were unstable across the study period (see Table 3). Given that the majority of participants remained in their baseline type or moved to *Collaborative & Helpful* as expected per the goals of FAMS, we moved forward with analyzing groups by type assigned at baseline.

**Table 3:** Stability of types across study period

	Stable in type assigned at baseline	Moved to and stable in Collaborative & Helpful	Moved to and stable in other type or unstable
Type	N (%)	N (%)	N (%)
Collaborative and helpful	15 (75%)	n/a	5 (25%)
Critically involved	7 (41%)	5 (29%)	4 (24%)
Satisfied with low involvement	10 (50%)	6 (30%)	4 (20%)
Want more involvement	8 (40%)	9 (45%)	3 (15%)
Total	40 (52%)	20 (26%)	16 (21%)

Individuals who were not consistently classified in a type were considered “unstable”. One individual who was *Critically Involved* at baseline did not complete at least 2 follow-up assessments and is excluded from this table which results in the totals equaling 99%

### Aim 1: Understand types from participants’ own experiences of family

There was evidence of the defining characteristics of types in participants’ descriptions of their experiences with family. *Collaborative & Helpful*, notable for high levels of helpful involvement, satisfaction, and collaboration, had a participant who described, “I like the fact that–I mean, I know I'm accountable for my own actions, but that little extra push from somebody, especially somebody that cares about you that loves you and doesn’t want to see anything bad happen to you, you’re going to always try and do what’s right versus what’s wrong” (54 year-old, non-Hispanic Black female). Another *Collaborative & Helpful* participant shared how his grandson, who has type 1 diabetes, taught him how to use a continuous glucose monitor, stating, “[my grandson] talked me through it, and he told me where to put it and how to place it and, you know, how to prep the site and when to push the button and said, ‘Don’t be scared, Granddaddy. You know it’s going to… It might hurt just a little bit, but it’s okay’… It was good for our family,” (70 year-old non-Hispanic white male).

*Critically Involved,* notable for high levels of helpful and harmful involvement, high criticism, and low effectiveness had a participant who said, “Well, my wife and daughter… I told my doc that they were getting on my nerves, harassing me, basically” (46 year-old, non-Hispanic Black male). Another *Critically Involved* participant stated their family member, “acts like she’s my mom, so she asks me like my mom would ask me. You know, you’re not–you’re not my mom… it was her tone of voice and the way she used it, not–maybe not necessarily the way she asked it. Just her tone of voice, it was like my mom. ‘How come you didn’t do it that way? You did great, but you could have done better’ (61 year-old non-Hispanic white female).

*Satisfied with Low Involvement,* notable for low levels of involvement and collaboration without dissatisfaction had a participant who described, “family involved in [diabetes]… while I understand how important that could be in certain circumstances, in my particular circumstance it wasn’t quite that important at all” (67 year-old, non-Hispanic white male). Another *Satisfied with Low Involvement* participant shared, “I really didn’t [interact with family/friends about diabetes]. If my kids asked me about it, I would tell them, but I was just kind of private about it, and I just didn’t talk about it with them. I didn’t share my A1c numbers. I didn’t share my blood sugar numbers when I was taking it. I just sort of went about my business” (62 year-old non-Hispanic white female).

Finally, *Want More Involvement,* notable for low involvement and collaboration with dissatisfaction had a participant who explained, “a large part of the reason I got into the study was because of my support person and because she hadn’t grown up around diabetics and didn’t realize on a day-to-day basis what was involved and what the challenges were” (55 year-old, non-Hispanic white male). Another *Want More Involvement* participant described how their family was aware of their diabetes but did not pay as much attention to it, sharing, “they knew it was there, but nobody knew how serious it was. So, it really wasn’t there. It was there, but it wasn’t. It was in the room, but it was on the–in the corner of the room,” (53 year-old, non-Hispanic white male).

### Aim 2: Descriptions of family experiences during the intervention by type at enrollment

We found themes Presence of Harmful Involvement, Increases in Communication*,* and Received Helpful Involvement were present within each type’s description of their experiences with family during FAMS, but there was notable variability across types in prevalence or dimensions (detailed in the paragraphs below). Presence of Harmful Involvement captured participants’ descriptions of specific harmful behaviors family engaged in such as nagging or making negative comments about their diabetes self-management choices or bringing certain foods into the home despite requests not to. When directly questioned, participants denied involvement that was “harmful;” however, upon reading and coding transcripts, we found examples of harmful behaviors described. Participants also noted Increases in Communication with their family about their diabetes and health across their intervention experience. This included both new conversations that had not been had before as well as inviting family into deeper conversations about diabetes. Finally, participants described Received Helpful Involvement, including both instrumental support (help with specific tasks or solving specific problems) and emotional support (having someone to non-judgmentally listen, discuss their diabetes, and encourage them). Specific similarities and differences by type are described below with quotations in Table 4.

**Table 4:** Exemplar quotations across types

	Presence of harmful involvement	Increases in communication	Received helpful involvement
Collaborative and helpful	“Then if [they] do remember I’m a diabetic, then they say no to the cake but then they have a massive bread type dinner. It’s not helpful… they would never in a, a million years try to hurt me. It was just, you know–I’m only allowed 30 g of carbs a meal.” (50 year-old, non-Hispanic, white female)	“It’s just a communication that’s been, it’s been amazing. Because it’s been hard. But it’s been good. And, you know, having him, listen to me… Having him help me. He’s been great. Really has. We’ve grown together.” (44 year-old, non-Hispanic white female) “We’re more open with each other now. We’ve always helped each other with meal planning and cooking and cleaning up the kitchen and kind of take equal shares of that. But now, I think it’s even more specific to what I may need as far as my diabetes is concerned. As far as what’s the most beneficial meal that we can have where it won’t jack your blood sugar up type thing.” (49 year-old, non-Hispanic white male)	“She didn’t boss me around by any means… So, [I] got a lot of encouragement from her.” (53 year-old, non-Hispanic white male) “She would be on that same eating plan with me–so it wouldn’t be like I got to eat a special way and she could eat whatever she wants. She would be doing the same thing so it would be easier for us to stay on track.” (54 year-old, non-Hispanic Black female)
Critically involved	“But, at the same time, they were not very supportive…I’m not a big sweets guy or anything, but if I wanted to eat some cookies or something, they'd say, ‘Okay. Here’s some cookies.’… My wife would make pasta quite often of some sort or something that has a lot of starch, carbs in it. And I would tell her, ‘Look I’m not going to eat much of that. And I told you before, I’m trying to cut that out,’ but it didn’t sink in.” (56 year-old, non-Hispanic white male) “My support person was my wife. But, if I went to go eat something, I couldn’t eat what I wanted to eat. ‘Cause she would be right there. And she was always watching over what I could do and what I couldn’t do.” (53 year-old, non-Hispanic white male)	“The tone…shifted from something that was harsher to something that was a little bit more open and understanding.” (64 year-old, non-Hispanic Asian Indian male) “I mean sometimes she’d fuss at me, but really I probably deserved it. But it was helpful. But it wasn’t unhelpful. It was helpful, but sometimes we might get into little arguments.” (65 year-old, non-Hispanic white male)	“She began to search, and I thought it was real[ly] neat–that she would, she was searching out recipes to try out, things she had not done before. And I thought that was beyond what she normally would have done… I think that was a needed step. You know, trying new things and searching out recipes that were appropriate.” (71 year-old, non-Hispanic white male)
Satisfied with low involvement	“I still feel like I get judged just a little bit, probably more now than I did before the program. But not in a bad way.” (51 year-old, non-Hispanic, white female) “I think more of my dad as…the parent…even though I’m 45 years-old, he’s still in that, “Are you doing what you’re supposed [to]?”… Now, I’m not saying he treats me like a child, but you know, he’s my dad. So… that’s kind of his view of me versus my friends who have a different view of me.” (44 year-old, non-Hispanic, white male)	[describing the helpful accountability provided by a friend around a self-care goal to consume less soda] “Any time I’m ever wanting a Coke, I text [name], and she says, ‘Don’t do it.’ So, just so you know, I’m almost 70 weeks free of soda. And she always tells me, ‘No, don’t do it.’ So, as I said before, [name] and I, the frequency in which we talked about health increased.” (42 year-old non-Hispanic white female) “I think throughout the program, I’ve learned to not only be able to discuss it [diabetes] but that, there’s just a lot of benefit to talking about things, and it’s not taboo…I did start discussing it with some coworkers. And I learned that some of them were in the exact same position, taking the exact same meds and dealing with the exact same thing and having to avoid the exact same foods. And, in learning how to discuss it, I learned how to relate to people more and to just talk more.” (46 year-old, non-Hispanic white male)	“I think it was probably both the support person and one of my daughters. We were able to have conversations when I was struggling or didn’t understand why glucose numbers were going up; there were people who were listening and not just making judgements or comments but really trying to hear and help me process. So, it was more relational than maybe functional or both.” (73 year-old, non-Hispanic white male)
Want more involvement	“[I am] criticized for my choices. I know I’m not supposed to have that candy bar, but when you tell me I can’t have that candy bar, or you belittle me because I want to have that candy bar, it just makes me want it more.” (54 year-old non-Hispanic, white female) “Because if I told them that I had diabetes and if I ate something wrong, some people would go, ‘Why is you eating that;’ you know? Try to embarrass you a little bit.” (67 year-old, non-Hispanic white male)	“Just being able to have that conversation with my children was a lot of it was because of the study that we engaged [in] and sharing that information with my children and everything. And, so, I think that all just as a family helped us to understand what we were dealing with.” (56 year-old, non-Hispanic Black female)	“Actually, it kind of really helped my son-kind of be more cognizant about asking me how I was doing, how my diabetes was, had I been walking.” (56 year-old, non-Hispanic black female) “He’s better about, uh, not bringing it [sugary foods] in. And, like I said before, if I asked for some salad fixings or whatever, he’s more likely to bring it home for me where six months ago he might not have.” (59 year-old, non-Hispanic white female)

### Collaborative & Helpful 

When *Collaborative & Helpful* discussed harmful involvement, their statements were qualified to give their family the benefit of the doubt by assuming this was due to family not being able to remember all the specifics of managing diabetes. *Collaborative & Helpful* described feeling closer to family during FAMS. Their descriptions of interacting with family were very collaborative, high in warmth, and suggested they trusted their family. They felt the topic of diabetes was less taboo to discuss or normalized through FAMS, allowing for specific conversations about their self-care and dietary needs with their families.

*Collaborative & Helpful* reported liking both emotional and instrumental support received during FAMS. Emotional support was described as ‘showing up’ for the individual with diabetes or being ‘in their corner’. Many mentioned family providing encouragement and checking in and/or paying attention to their diabetes-related needs. Instrumental support proffered was described as aligning with their needs; examples included walking together, medication reminders, and food preparation.family

### Critically Involved

*Critically Involved* provided examples of harmful involvement included family nagging, bringing ‘sweets’ into the home, and policing what they were eating. Some *Critically Involved* reported that their conversations with family had a deeper ‘level’ or better ‘tone’ than before FAMS; however, several examples provided suggested the support was still more controlling than autonomy supportive throughout the intervention. Participants in this type also noted that it was easier to talk about diabetes due to FAMS.

*Critically Involved* emphasized instrumental support over emotional support experienced during FAMS. Common examples of instrumental support included cooking diabetes-friendly foods for the family and meal planning, medication reminders, and taking walks together. Less common examples of instrumental support including helping with self-care goals, acquiring medical supplies, and using a continuous glucose monitor (CGM) together. Emotional support was mostly in the form of showing concern for the individual with diabetes and being more informed about their condition.

### Satisfied with Low Involvement

*Satisfied with Low Involvement*, like other types, were hesitant to directly discuss harmful involvement from family. The minority who did described their family bringing home ‘sweets’ and being aware of their dietary needs but not changing their behavior in response. Throughout FAMS, *Satisfied with Low Involvement* described sharing their experience with diabetes with family more frequently and in return receiving encouragement and support. Additionally, *Satisfied with Low Involvement* found that as they opened up to their family about their diabetes, the shame and stigma they felt around the disease decreased and the support they were able to access increased. Participants described these new conversations as a safe place to land that historically may not have felt as welcoming.

Finally, *Satisfied with Low Involvement* emphasized emotional support over instrumental support. Emotional support was described as opening new conversations and using family as ‘sounding boards.’ Similar changes in family behaviors were noted (e.g. food choices, walking together) but the person with diabetes seemed less enthusiastic about these changes than *Critically Involved*.

### Want More Involvement

*Want More Involvement* described their family as being harmful by critiquing them and embarrassing them for how they were managing their diabetes. *Want More Involvement* described sharing more information with their families and having conversations they had never had before during FAMS. One described developing an accountability partner relationship where they shared goals and kept each other motivated. *Want More Involvement* reported equally liking emotional and instrumental support. There were different types of emotional support described including heightened awareness of their diabetes-related needs, checking in, and accountability. For instrumental support, reminders were very common, walking together was mentioned, and a few expressed desires for more instrumental support than experienced.

## Discussion

This study qualitatively explored the utility of the recently developed typology of diabetes-specific family functioning in understanding participants’ experiences with family during an intervention seeking to improve family involvement in diabetes self-management. Participants’ descriptions of their family experiences reflected the characteristics of the type assigned with the quantitative survey typology assessment tool, lending face validity to the typology. We found consistent themes across types and differences between types in their experiences with family during the intervention. Across types, harmful involvement was present and adults with T2D were hesitant to label it as such. Communication about diabetes and health increased during FAMS, and adults with T2D received more emotional and instrumental support from their families.

Areas of difference across types within the themes are of particular note. Within the first theme of Presence of Harmful Involvement, there were few differences across types; however, *Critically Involved* more readily mentioned harmful involvement when asked than other types. Additionally, *Satisfied with Low Involvement* were less ready to excuse or explain away harmful behaviors than *Collaborative & Helpful*. In the theme of Increases in Communication*, Critically Involved* and *Collaborative & Helpful* described how it was easier to talk about diabetes with friends and family; however, only *Collaborative & Helpful* used these conversations to ask for specific support. *Satisfied with Low Involvement* and *Want More Involvement* both described sharing things about their diabetes with their friends and family they previously had not shared. Regarding Received Helpful Involvement, emotional support was more pronounced for *Satisfied with Low Involvement* while instrumental support was more pronounced for *Critically Involved.* Both *Collaborative & Helpful* and *Want More Involvement* endorsed emotional and instrumental support, and *Want More Involvement* was the only type to emphasize the importance of reminders from family and friends.

This is the first application of the typology to understand experiences with an intervention qualitatively and it sheds light on areas for future research, particularly regarding intervention tailoring for each type. Results indicate different types may have different intervention needs. *Collaborative & Helpful* really enjoyed the intervention’s focus on involving family. Family was highly involved in helpful and autonomy supportive ways for *Collaborative & Helpful*. In FAMS, most of the intervention focused on the adult with T2D. Family and friends received text messages but were not involved in coaching calls or goal setting. Due to the positive and collaborative relationship with family that is typical of *Collaborative & Helpful*, a dyadic intervention more fully involving family may be welcomed and appropriate.

We expected FAMS’s focus on relational aspects such as assertive communication training to decrease criticism and be a good fit for *Critically Involved.* Unfortunately, *Critically Involved* reported persistent issues with harmful involvement and their descriptions of family included controlling aspects. It’s possible that interventions that seek to engage family–even when addressing harmful aspects–may not be a good fit on average for *Critically Involved*. Rather interventions that do not engage family in self-management or interventions that train family members directly on communication may be more successful.

We were surprised by how much *Satisfied with Low Involvement* liked FAMS, and so were they. Data demonstrated more positive experiences with the intervention than negative; however, there were some adults with this type who maintained their individual approach versus a family approach to the management of their disease despite participation in FAMS. *Satisfied with Low Involvement* were more focused on emotional than instrumental support. Disease-focused actions may have felt intrusive or like they were being ‘treated as children,’ as has been described elsewhere (Mayberry & Osborn, 2012). Instead, *Satisfied with Low Involvement* comments oriented towards increasing emotional support.

Finally, there was good fit between FAMS and *Want More Involvement.* This type described how FAMS helped their families change their behaviors in order to meet their needs. Interestingly, family providing reminders for self-care was frequently described as a positive by *Want More Involvement*, and it is unclear why reminders were so salient for this group. It is possible that due to the higher cognitive compensation needs typical of this group (Mayberry et al., 2021, 2023) that reminders provided a strong match between their desired and received involvement. Alternatively, *Want More Involvement* is also characterized by lower helpful involvement (Mayberry et al., 2021, 2023), and family beginning to be involved at all, by providing reminders, is a welcome change.

Qualitative findings provide nuance to quantitative findings on the typology. For instance, both *Want More Involvement* and *Satisfied with Low Involvement* are characterized by low helpful involvement (Mayberry et al., 2021, 2023), but here we learned the former thrived with increased instrumental support while the later preferred emotional support. Future interventions could investigate deepening family involvement over time for *Want More Involvement*–starting with providing reminders and building to doing the behavior or activity together. Harmful aspects of family involvement, assessed quantitatively, are often experienced by adults with T2D (Mayberry & Osborn, 2012, 2014). However, interviewed participants were hesitant to describe any behavior as “harmful”, even with direct questioning, making it difficult to draw firm conclusions about how the intervention may have impacted harmful aspects. Although there were similarities in harmful involvement experienced across types, there may have been differences in how types responded to harmful involvement as well. Specifically, *Critically Involved, Satisfied with Low Involvement,* and *Want More Involvement* all discussed feeling judged or guilty for their choices, possibly internalizing the harmful comments. On the other hand, while *Satisfied with Low* felt frustrated with their families who continued to bring unhelpful foods into the home, *Collaborative & Helpful* gave family the benefit of the doubt in similar situations. Future research could explore how existing family dynamics influence responses to harmful behaviors.

## Limitations and future directions

Participants experienced a family-focused mobile delivered intervention which likely biased some of their responses compared to a sample who had not. There may be less variability in responses across types because they were all exposed to FAMS. Thus, the type differences in comments about family are especially striking. Participants were retrospectively reporting on experiences from 6–15 months prior to the interviews (covering experiences before, during and after their 9 month intervention experience) which may have hampered recall. However, the rich descriptions and examples provided in the interviews suggest the experiences were meaningful and salient. We grouped participants based on their baseline type for analyses, knowing that type changes over time (Mayberry et al., 2023). Had we conducted interviews at baseline or over the course of the intervention, we may have found different themes. Similarly, there may be type differences in sections of the interview not coded here.

## Conclusions

Qualitative data fills in the gaps of quantitative data to create a fuller picture by including participants’ perspectives and elucidating differences and nuances not evident in quantitative data. Here, we added qualitative validity to a typology that had previously been quantitatively validated (Mayberry et al., 2021, 2023). Our findings provide support for the idea that family interventions for diabetes management should not be a “one-size-fits-all” approach given the diversity in family functioning and discrepancies between individuals’ desired and received involvement. Opportunities exist to use this typology to begin to match proffered interventions to individual needs, which will ultimately make interventions more effective.

## Data Availability

Data is available from the corresponding author by reasonable request.

## References

[CR1] Baig, A. A., Benitez, A., Quinn, M. T., & Burnet, D. L. (2015). Family interventions to improve diabetes outcomes for adults. *Annals of the New York Academy of Sciences,**1353*(1), 89–112. 10.1111/nyas.12844 26250784 10.1111/nyas.12844 PMC4624026

[CR2] Berg, C. A., Schindler, I., Smith, T. W., Skinner, M., & Beveridge, R. M. (2011). Perceptions of the cognitive compensation and interpersonal enjoyment functions of collaboration among middle-aged and older married couples. *Psychology and Aging,**26*(1), 167. 10.1037/a0021124 20973607 10.1037/a0021124

[CR3] Braun, V., & Clarke, V. (2006). Using thematic analysis in psychology. *Qualitative Research in Psychology,**3*(2), 77–101. 10.1191/1478088706qp063oa

[CR4] Fort, M. P., Steiner, J. F., Santos, C., Moore, K. R., Villaverde, M., Nease, D. E., Ortega, D., & Manson, S. M. (2020). Opportunities, challenges, and strategies for engaging family in diabetes and hypertension management: A qualitative study. *Journal of Health Care for the Poor and Underserved,**31*(2), 827–844. 10.1353/hpu.2020.0063 33410810 10.1353/hpu.2020.0063

[CR5] Karlsen, B., & Bru, E. (2014). The relationship between diabetes-related distress and clinical variables and perceived support among adults with type 2 diabetes: A prospective study. *International Journal of Nursing Studies,**51*(3), 438–447. 23891535 10.1016/j.ijnurstu.2013.06.016

[CR6] Lee, A. A., Piette, J. D., Heisler, M., & Rosland, A.-M. (2018). Diabetes distress and glycemic control: The buffering effect of autonomy support from important family members and friends. *Diabetes Care,**41*(6), 1157–1163. 29599295 10.2337/dc17-2396 PMC5961390

[CR7] Leukel, P. J., Kollin, S. R., Lewis, B. R., & Lee, A. A. (2022). The influence of emotion regulation and family involvement on diabetes distress among adults with type 2 diabetes. *Journal of Behavioral Medicine,**45*(6), 904–913. 10.1007/s10865-022-00351-0 35948697 10.1007/s10865-022-00351-0 PMC9364847

[CR8] QSR International Pty Ltd. (2018). *NVivo* Version 12. https://www.qsrinternational.com/nvivo-qualitative-data-analysis-software/home

[CR9] Mayberry, L. S., Berg, C. A., Greevy, R. A., Jr., & Wallston, K. A. (2019). Assessing helpful and harmful family and friend involvement in adults’ type 2 diabetes self-management. *Patient Education and Counseling,**102*(7), 1380–1388. 10.1016/j.pec.2019.02.027 30922622 10.1016/j.pec.2019.02.027 PMC6546510

[CR10] Mayberry, L. S., El-Rifai, M., Nelson, L. A., Parks, M., Greevy, R. A., Jr., LeStourgeon, L., Molli, S., Bergner, E., Spieker, A., & Aikens, J. E. (2022). Rationale, design, and recruitment outcomes for the family/friend activation to motivate self-care (FAMS) 2.0 randomized controlled trial among adults with type 2 diabetes and their support persons. *Contemporary Clinical Trials,**122*, Article 106956. 36208719 10.1016/j.cct.2022.106956 PMC10364455

[CR11] Mayberry, L. S., Greevy, R. A., Huang, L.-C., Zhao, S., & Berg, C. A. (2021). Development of a typology of diabetes-specific family functioning among adults with type 2. *Annals of Behavioral Medicine,**55*(10), 956–969. 10.1093/abm/kaab009 33761527 10.1093/abm/kaab009 PMC8489307

[CR12] Mayberry, L. S., & Osborn, C. Y. (2012). Family support, medication adherence, and glycemic control among adults with type 2 diabetes. *Diabetes Care,**35*(6), 1239–1245. 10.2337/dc11-2103 22538012 10.2337/dc11-2103 PMC3357235

[CR13] Mayberry, L. S., & Osborn, C. Y. (2014). Family involvement is helpful and harmful to patients’ self-care and glycemic control. *Patient Education and Counseling,**97*(3), 418–425. 10.1016/j.pec.2014.09.011 25282327 10.1016/j.pec.2014.09.011 PMC4254324

[CR14] Mayberry, L. S., Zhao, S., Roddy, M. K., Spieker, A. J., Berg, C. A., Nelson, L. A., & Greevy, R. A. (2023). Family typology for adults with type 2 diabetes: Longitudinal stability and predictive validity for diabetes management and well-being. *Diabetes Care*. 10.2337/dc23-0827 37708437 10.2337/dc23-0827 PMC10620540

[CR15] Nelson, L. A., Spieker, A. J., Greevy, R. A., Roddy, M. K., LeStourgeon, L. M., Bergner, E. M., El-Rifai, M., Aikens, J. E., Wolever, R. Q., Elasy, T. A., & Mayberry, L. S. (2023). Glycemic outcomes of a family-focused intervention for adults with type 2 diabetes: Main, mediated, and subgroup effects from the FAMS 2.0 RCT. *Diabetes Research and Clinical Practice,**206*, 110991. 10.1101/2023.09.11.23295374 37925077 10.1016/j.diabres.2023.110991 PMC10873034

[CR16] Nicklett, E. J., Heisler, M. E. M., Spencer, M. S., & Rosland, A.-M. (2013). Direct social support and long-term health among middle-aged and older adults with type 2 diabetes mellitus. *The Journals of Gerontology Series b: Psychological Sciences and Social Sciences,**68*(6), 933–943. 24150176 10.1093/geronb/gbt100 PMC3805290

[CR17] Roddy, M. K., Spieker, A. J., Nelson, L. A., Greevy, R. A., LeStourgeon, L. M., Bergner, E. M., El-Rifai, M. W., Elasy, T. A., Aikens, J. E., Wolever, R. Q., & Mayberry, L. S. (2023). Well-being outcomes of a family-focused intervention for persons with type 2 diabetes and support persons: Main, mediated, and subgroup effects from the FAMS 2.0 RCT. *Diabetes Research and Clinical Practice,**204*, 110921. 10.1016/j.diabres.2023.110921 37742801 10.1016/j.diabres.2023.110921 PMC10617415

[CR18] Roddy, M. K., Spieker, A. J., Greevy, R. A., Jr., Nelson, L. A., Berg, C., & Mayberry, L. S. (2025). Diabetes-specific family functioning typology associated with intervention engagement and effects: Secondary analyses from a randomized controlled trial. *Annals of Behavioral Medicine,**59*(1), Article kaae070. 39661957 10.1093/abm/kaae070 PMC11783318

[CR19] Rosland, A.-M., Heisler, M., & Piette, J. D. (2012). The impact of family behaviors and communication patterns on chronic illness outcomes: A systematic review. *Journal of Behavioral Medicine,**35*(2), 221–239. 10.1007/s10865-011-9354-4 21691845 10.1007/s10865-011-9354-4 PMC3785075

[CR20] Song, Y., Song, H.-J., Han, H.-R., Park, S.-Y., Nam, S., & Kim, M. T. (2012). Unmet needs for social support and effects on diabetes self-care activities in Korean Americans with type 2 diabetes. *The Diabetes Educator,**38*(1), 77–85. 22222514 10.1177/0145721711432456 PMC3649548

[CR21] Strauss, A., & Corbin, J. (1990). Basics of qualitative research (Vol. 15, pp. 61−110). Newbury Park, CA: sage.

[CR22] Tang, T. S., Brown, M. B., Funnell, M. M., & Anderson, R. M. (2008). Social support, quality of life, and self-care behaviors among African Americans with type 2 diabetes. *The Diabetes Educator,**34*(2), 266–276. 10.1177/0145721708315680 18375776 10.1177/0145721708315680

[CR23] Torenholt, R., Schwennesen, N., & Willaing, I. (2014). Lost in translation—The role of family in interventions among adults with diabetes: A systematic review. *Diabetic Medicine,**31*(1), 15–23. 23870045 10.1111/dme.12290

[CR24] Williams, G. C., Freedman, Z. R., & Deci, E. L. (1998). Supporting autonomy to motivate patients with diabetes for glucose control. *Diabetes Care,**21*(10), 1644–1651. 9773724 10.2337/diacare.21.10.1644

[CR25] Zhang, H., Zhang, Q., Luo, D., Cai, X., Li, R., Zhang, Y., Lu, Y., Liu, J., Gu, J., & Li, M. (2022). The effect of family-based intervention for adults with diabetes on HbA1c and other health-related outcomes: Systematic review and meta-analysis. *Journal of Clinical Nursing,**31*(11–12), 1488–1501. 34888968 10.1111/jocn.16082

